# Gilded nanoparticles for plasmonically enhanced fluorescence in TiO_2_:Sm^3+^ sol-gel films

**DOI:** 10.1186/1556-276X-9-143

**Published:** 2014-03-25

**Authors:** Siim Pikker, Leonid Dolgov, Siim Heinsalu, Sergii Mamykin, Valter Kiisk, Sergei Kopanchuk, Rünno Lõhmus, Ilmo Sildos

**Affiliations:** 1 Institute of Physics, University of Tartu, Riia 142, Tartu 51014, Estonia; 2 V. Lashkaryov Institute of Semiconductor Physics of National Academy of Sciences of Ukraine, Kyiv 03680, Ukraine; 3 Institute of Chemistry, University of Tartu, Ravila 14A, Tartu 50411, Estonia

**Keywords:** Silica-gold core-shell nanoparticles, Plasmonics, Rare-earth fluorescence, Metal-enhanced fluorescence

## Abstract

**Abstract:**

Silica-gold core-shell nanoparticles were used for plasmonic enhancement of rare earth fluorescence in sol-gel-derived TiO_2_:Sm^3+^ films. Local enhancement of Sm^3+^ fluorescence in the vicinity of separate gilded nanoparticles was revealed by a combination of dark field microscopy and fluorescence spectroscopy techniques. An intensity enhancement of Sm^3+^ fluorescence varies from 2.5 to 10 times depending on the used direct (visible) or indirect (ultraviolet) excitations. Analysis of fluorescence lifetimes suggests that the locally stronger fluorescence occurs because of higher plasmon-coupled direct absorption of exciting light by the Sm^3+^ ions or due to plasmon-assisted non-radiative energy transfer from the excitons of TiO_2_ host to the rare earth ions.

**PACS:**

78; 78.67.-n; 78.67.Bf

## Background

Noble metal nanoparticles are under intense scientific and applied attention because of their unique optical properties [1]. Incident light which is in resonance with the collective electronic oscillations near the surface of metal nanoparticles causes the so-called localized surface plasmon resonance. It results in strong concentration of light energy and electric field in the subwavelength nanoscale region near the particle. The strong local field causes an increase in the efficiency of light absorption, scattering, and fluorescence [2].

Metal-enhanced fluorescence as a branch of nano-optics was formed on the one hand from the needs of fluorescent sensing of minute amounts of matter [2,3] and on the other hand from fundamental interest to the control of light energy on the nanoscale and inducing of coherent plasmons with low damping [4]. Effective coupling of plasmons with fluorescent light is actual also for the fluorescent glasses [5,6] and active optical waveguides [7]. Trivalent rare earth (RE) ions, which are popular due to their efficient narrow-band photostable fluorescence, are of special interest as subjects for plasmonic enhancement. It is because their absorption cross sections as well as radiative decay rate are both very low compared to other emitters, such as dye molecules. There are a few studies suggesting local plasmonic enhancement of RE fluorescence induced by noble metal nanodopant in sol-gel-derived optical materials, such as silica glasses and active fibers in the visible [5,6] and infrared [7] spectral ranges. Yet, the preparation of such samples requires specific methods for dispersion of metal particles in the host media, avoiding their aggregation and oxidation, especially for the silver nanoparticles [6,8]. As far as we know, detected local enhancement of fluorescence intensity in the RE-doped sol-gel materials does not exceed two to three times [5-7]. Plasmonic resonance in small metal particles (approximately 5 to 20 nm) mainly causes a waste of the incident light energy as heat and do not contribute significantly to fluorescence enhancement. In contrast, plasmonic resonance in bigger nanoparticles (>50 nm) results in a stronger light scattering, which could support fluorescence more essentially in the resonance spectral range [3]. However, the synthesis of such bigger nanoparticles with uniform size is not an easy task.

Hereby, we propose to utilize silica-gold core-shell nanoparticles described earlier by Pham et al. [9] for the enhancement of RE^3+^ fluorescence. Relatively big uniform size of silica core approximately 140 nm together with the plasmonic properties of the gold shell provide the necessary conditions for the plasmonically enhanced fluorescence which will be demonstrated in the case of sol-gel-derived TiO_2_:Sm^3+^. Used delicate combination of microscopic and spectroscopic techniques allowed investigation of Sm^3+^ fluorescence in the vicinity of separate gilded nanoparticles and detection of up to 10 times higher local intensity of emitted light.

## Methods

Silica core nanoparticles were prepared by Stöber method [10] and functionalized by amino groups providing good covering of the silica core by the gold seeds. Then, joining of the gold seeds and formation of a continuous gold shell around the silica core were realized [9]. Gilded nanoparticles dispersed in water were obtained. Plasmonic light extinction by this dispersion was confirmed by using Jasco V-570 spectrophotometer (Easton, MD, USA). The gilded nanoparticles were redispersed (approximately 0.6 wt.%) in butanol and added into the titanium butoxide precursor containing 2 mol% of samarium salt. This mixture was spin-coated on the glass substrates and annealed at 500°C. Thus, TiO_2_:Sm^3+^ films doped with gilded nanoparticles were obtained.

Optical imaging and microluminescence measurements were carried out on a home-assembled setup based on Olympus BX41M microscope (Olympus Corporation, Shinjuku-ku, Japan) combined with Andor iXon electron multiplying charge coupled device (EMCCD) camera (Springvale Business Park, Belfast, UK ) for highly sensitive optical imaging and fiber-coupled Andor SR303i spectrometer with Andor Newton camera for spectral measurements. Colored image of light scattering from bigger sample area was made by digital photocamera attached to an ocular of the microscope because the EMCCD camera used for fluorescence imaging has only black and white mode. Both dark field and fluorescence measurements were carried out by using a side illumination. In the case of dark field imaging, the beam of a bright white light-emitting diode (LED) was used so that the field of view remains dark if no scattering entities were present in the sample. The fluorescence was excited with a 355 nm diode-pumped solid-state (DPSS) laser while the signal was observed though a long-pass filter. In the latter case, the small aperture of the single-mode fiber allowed highly confocal spectral measurements in spite of the wide-field illumination. Alternatively, spectral measurements with point excitation were possible by using 532 nm DPSS laser focused onto the sample through the microscope objective.

Fluorescent lifetimes were measured by multichannel analyzer P7882 (FAST ComTec, München, Germany) connected to the photomultiplier. Also, we have determined fluorescence lifetimes in the time-gating luminescence mode (TGL) using an imaging attachment (LIFA-X, Lambert Instruments, Roden, The Netherlands) consisting of a signal generator, multi-LED excitation source with a 3-W LED (532 nm) and an intensified charge coupled device (CCD) Li^2^CAM-X with GEN-III GaAs photocathode. The CCD was mounted on the side port of an iMIC inverted digital fluorescence microscope (Till Photonics GmbH, Gräfelfing, Germany) through a TuCam adapter with × 2 magnification (Andor Technology). Multi-LED was fiber-coupled to the epicondenser of iMIC. The filter cube comprised of a BrightLine HC 520/35 nm (Semrock, Rochester, NY, USA) exciter, a Zt 532 rdcxt dichroic (Chroma, Bellows Falls, VT, USA) and ET 605/70 M nm (Chroma) emitter. Photons were collected with × 4 UPLSAPO objective (Olympus, Shinjuku-ku, Japan). Camera binning of 4 × 4 was used. In TGL mode, the delay time between excitation pulses (for 10 μs) trigger off and camera gain trigger on (for 10 μs) was varied in the interval between 0.6 and 275 μs at cycle frequency of 3 kHz. Full camera exposure time per image was 300 ms. Obtained image data analysis was performed using Lambert instrument fluorescence lifetime imaging microscope (Li-FLIM v1.2.22) software.

## Results and discussion

**Figure 1:**
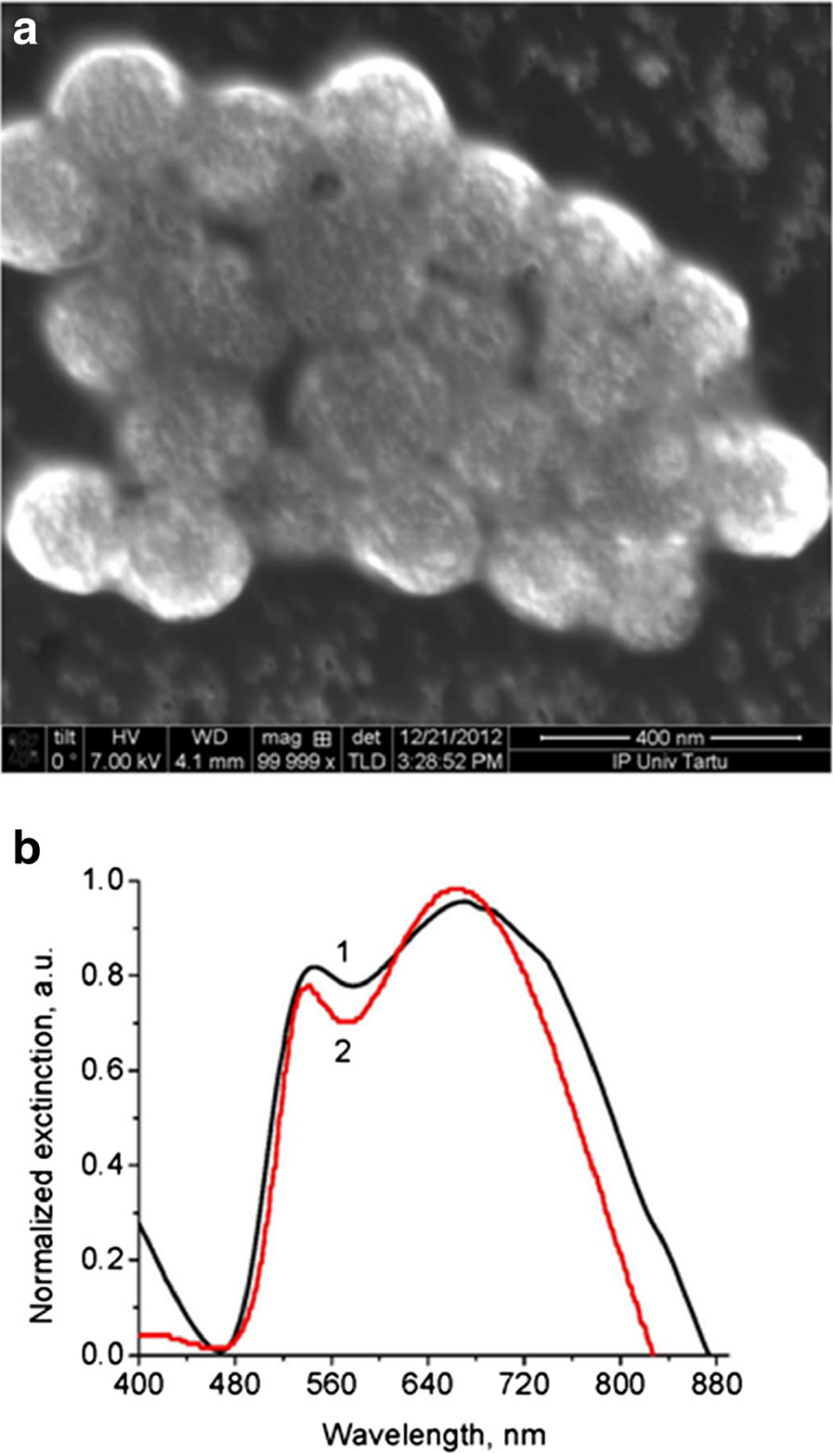
SEM image (a) and light extinction spectra (b) of spherical gilded nanoparticles.

Silica-gold core-shell nanoparticles were initially prepared as dispersion in water. For scanning electron microscopy (SEM) characterization, the droplets of this dispersion were deposited on a silicon substrate and dried. SEM images indicate globules with a narrow size distribution (Figure 1a). The size of silica core approximately 140 nm and thickness of the gold shell approximately 15 to 20 nm were estimated on the basis of several SEM images. Plasmonic properties of these nanoparticles become apparent already during the synthesis process because the spectrally selective plasmonic light absorption lead to a bluish color of the prepared dispersion. Light extinction spectra measured for the 1-cm layer of this dispersion consists of two bands with maxima at 525 and 675 nm (Figure 1b, curve 1). The shapes of these bands are related respectively to the quadrupole and dipole plasmonic resonances calculated according to the Mie theory (Figure 1b, curve 2).

**Figure 2:**
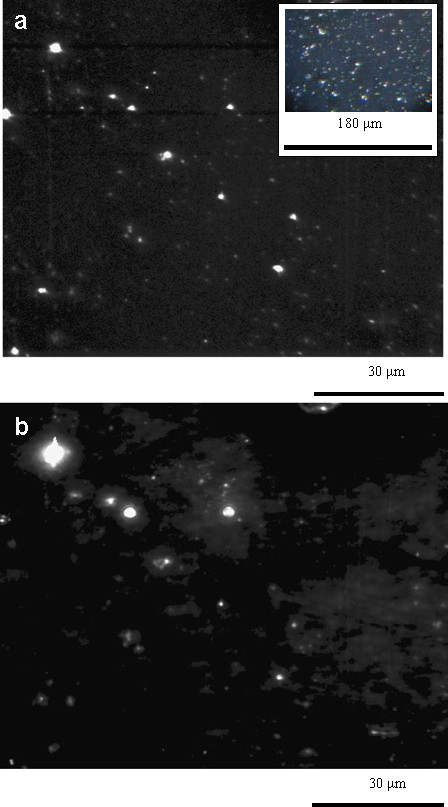
**Grayscale images of dark field light scattering (a) and fluorescence (b) from the TiO**_**2**_**:Sm**^**3+**^-**Au film (*λ***_**exc**_ **= 355 nm).**

In the dark field, optical images the single gilded nanoparticles look like colored spots on the dark background because of plasmonic light scattering (inset of Figure 2a). The corresponding fluorescence image under UV excitation shows bright red spots due to fluorescent Sm^3+^ ions on the uniform fluorescent background. Generally, there is an excellent correspondence between the spots detected in dark-field scattering (Figure 2a) and those observed in fluorescence (Figure 2b). In contrast, in the similarly prepared samples without gold co-doping, no bright spots were observed in fluorescence. This is a strong evidence about the plasmonic enhancement of Sm^3+^ fluorescence near the gilded nanoparticles.

**Figure 3:**
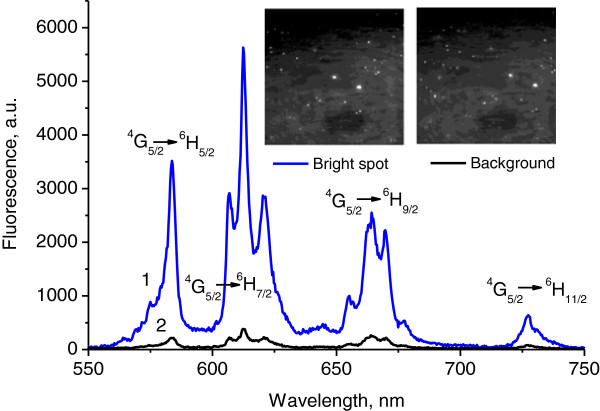
**Micro-luminescence spectra of TiO**_**2**_**:Sm**^**3+ **^**films doped with gilded nanoparticles: (1) bright spot, (2) background (*λ***_**exc**_ **= 355 nm).**

The fluorescence spectrum under ultraviolet 355 nm excitation is typical for Sm^3+^ ions in crystalline anatase-type TiO_2_ surrounding as evidenced by the crystal-field splitting of the four spectral bands due to the transitions ^4^G_5/2_ → ^6^H_5/2_, ^4^G_5/2_ → ^6^H_7/2_, ^4^G_5/2_ → ^6^H_9/2_, ^4^G_5/2_ → ^6^H_11/2_ of f electrons in Sm^3+^ ions. The intensity of the fluorescence at the bright spots near gilded nanoparticles is approximately 10 times higher than the background fluorescence of Sm^3+^ ions distant from metal inclusions (Figure 3).

Plasmonic enhancement of fluorescence is usually explained either by enhancement of light absorption or enhancement of radiative decay rate [1]. In the case of TiO_2_, at least two different RE excitation mechanisms must be distinguished. First mechanism is realized when the absorption of ultraviolet light causes intrinsic excitations in TiO_2_ host, such as self-trapped or impurity-trapped excitons. These excitons can non-radiatively transfer energy to the fluorescent impurity. The effective cross section of such indirect Sm^3+^ excitation is several orders of magnitude higher than direct absorption cross section 10^−21^ to 10^−20^ cm^2^ of Sm^3+^ ions for the visible light [11]. But ultraviolet light cannot efficiently excite plasmon in the gilded nanoparticles due to the lack of resonance. So, the reasons for the enhancement of Sm^3+^ fluorescence are either plasmonic enhancement of radiative decay rate or plasmonically assisted energy transfer from the excitons to the Sm^3+^ ions.

**Figure 4:**
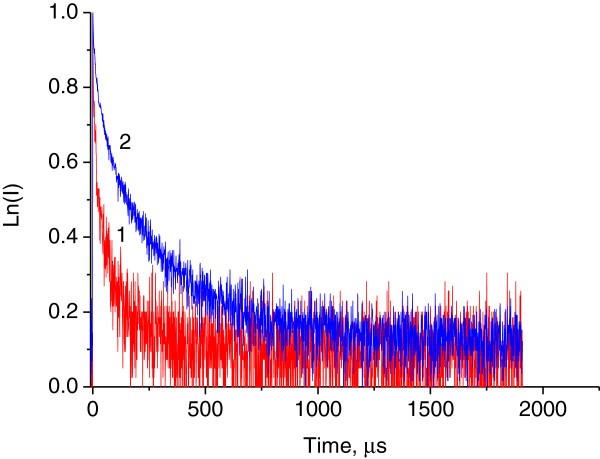
**Normalized experimental fluorescence decay kinetics: from background (1), from bright spot (2) of TiO**_**2**_**:Sm**^**3+**^-**Au films.**

Fluorescent decay rate is inversely proportional to the fluorescent lifetime. To check plasmonic influence on the decay rate, we measured the fluorescent kinetics for the bright spots and for the background rare earth fluorescence at the ultraviolet excitation *λ*_exc_ = 355 nm (Figure 4). It was necessary to use up to three exponential decay components to satisfactorily model the kinetics:

(1) $$\begin{array}{l} I\left(t\right)=A_{1}\exp\left(-\frac{t}{\tau_{1}}\right)+A_{2}\exp\left(-\frac{t}{\tau_{2}}\right)\\ \;+A_{3}\exp\left(-\frac{t}{\tau_{3}}\right), \end{array}$$

where *A*_1_, *A*_2_, and *A*_3_ are the coefficients of light intensity, *τ*_1_, *τ*_2_, *τ*_3_ are the lifetimes of fluorescence. In such situation, the overall rate of decay is frequently characterized by the average lifetime defined as

(2) $$\langle \tau \rangle =\frac{\int_{0}^{\infty }\mathrm{tI}\left(t\right)\mathrm{dt}}{\int_{0}^{\infty }I\left(t\right)\;\mathrm{dt}}=\frac{\sum_{i}A_{i}{\tau_{i}}^{2}}{\sum_{i}A_{i}\tau_{i}}$$

Obtained lifetimes of fluorescence are in the range of tens and hundreds of microseconds (Table 1). Fluorescence lifetimes of the order of hundreds of microseconds are typical for the rare earth ions situating in a good crystalline TiO_2_ anatase host [11]. Lifetimes in the range of tens of microseconds can be caused by Sm^3+^ fluorescent centers situating in the areas of TiO_2_ host having locally different crystallinity or local lattice defects. Corresponding lifetime components for the bright spots and for the background Sm^3+^ fluorescence are not very different. Based on this, we can suppose that the radiative rate of rare earth fluorophore is not very strongly influenced by localized plasmons. Detected approximately 10 times enhancement in the intensity of Sm^3+^ fluorescence at the ultraviolet excitation could be caused by plasmonic support of energy transfer from exciton to rare earth ions. Possibility of non-radiative plasmonic support for the excitons was recently demonstrated in the case of plasmonically improved photocatalysis [12]. Plasmonic support of Förster resonance energy transfer for quantum dot's fluorescence was described in [13].

**Table 1:** **Lifetimes of fluorescence for the TiO**_**2**_**:Sm**^**3+ **^**film doped with gilded nanoparticles, *λ***_**exc**_ **= 355 nm**

**Place on the sample**	***τ***_**1, **_**μs**	***τ***_**2**_**, μs**	***τ***_**3**_**, μs**	***τ*, μs**
Bright spot 1	2.4	25	156	103
Bright spot 2	6.5	48	299	147
Bright spot 3	10.5	78	294	202
Spot 1 on the background	4.1	35.3	225	138
Spot 2 on the background	7.4	50	220	137

**Figure 5:**
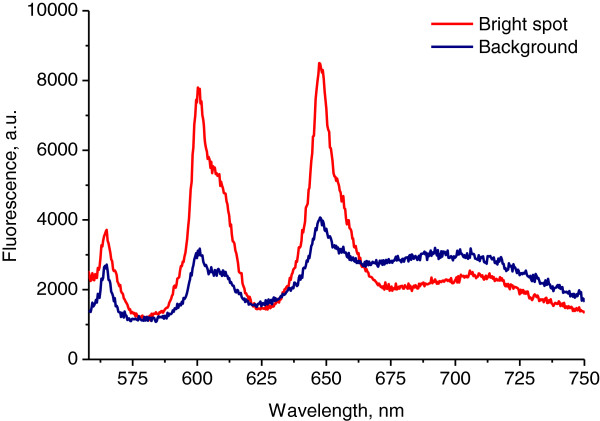
**Micro-luminescence spectra of TiO**_**2**_**:Sm**^**3+ **^**films doped with gilded nanoparticles: (1) bright spot, (2) background (*λ***_**exc**_ **= 532 nm).**

Excitation by green light, *λ*_exc_ = 532 nm, results in direct excitation of Sm^3+^ and also yields a fluorescence spectrum consisting of the four bands. But in this case, the bands are broader and almost featureless (Figure 5). It means that different ensemble of Sm^3+^ ions is excited in this case. The absence of spectral features suggests that those Sm ions are situated in less ordered TiO_2_ environment [14]. In spite of the exclusion of excitonic influence at such excitation, we detected still 2.5 times enhancement of fluorescence in the vicinity of gilded nanoparticles (Figure 5). Under 532 nm excitation, the Stokes shift of the fluorescence emission is very small [15]. So, both excitation and emission can be influenced by plasmons.

Fluorescence lifetimes at 532 nm excitation were measured in the time-gated mode on a FLIM in the spectral range of 580 to 660 nm. Obtained fluorescence decay is also multiexponential because different Sm^3+^ centers situate in TiO_2_ environment with different local surroundings. Numerical values of the lifetimes are similar to those presented in Table 1. Because of the insignificant changes in the lifetimes of Sm^3+^ fluorescence, we suppose that the detected 2.5 times enhancement in the intensity of fluorescence could be caused mainly by plasmon-enhanced direct absorption of exciting light by Sm^3+^ ions near the gilded nanoparticles.

## Conclusions

Silica-gold core-shell nanoparticles were synthesized and successfully adjusted for the incorporation into TiO_2_:Sm^3+^ films. Prospective capabilities of these particles for the local plasmonic enhancement of rare earth fluorescence are demonstrated. Detected locally strong Sm^3+^ fluorescence is connected more with local increase in light absorption and energy transfer than with changes in radiative decay rates since fluorescent lifetimes are not changed significantly. Detected enhancement of fluorescence can be based both on the plasmonic enhancement of direct light absorption by Sm^3+^ ions and on profitable plasmonic support of energy transfer from exciton to rare earth ions in the case of the indirect excitation. As a next step, variation of dielectric core and noble metal shell sizes can be used for the spectral tuning of the plasmon resonance and estimation of its impact on the plasmon-enhanced fluorescence.

## Competing interests

The authors declare that they have no competing interests.

## Authors’ contributions

SP, LD, and SH developed the idea of the work and participated in the preparation of sol-gel TiO_2_ samples activated by Sm^3+^ ions and in their doping by core-shell nanoparticles. SM synthesized silica-gold core-shell nanoparticles. VK and SK provided necessary fluorescent and microscopic measurements of the samples. RL made contribution to the revised version of the manuscript. SP realized scanning electron microscopy of the samples and proposed fruitful ideas for explanation of obtained results. IS participated in joint discussions of co-authors and in explanation of scientific results. All authors read and approved the final manuscript.
